# Single-step One-pot Synthesis of Graphene Foam/TiO_2_ Nanosheet Hybrids for Effective Water Treatment

**DOI:** 10.1038/srep43755

**Published:** 2017-03-02

**Authors:** Weilin Wang, Zhaofeng Wang, Jingjing Liu, Zhengguo Zhang, Luyi Sun

**Affiliations:** 1 Ministry of Education Key Laboratory of Enhanced Heat Transfer & Energy Conservation, School of Chemistry and Chemical Engineering, South China University of Technology, Guangzhou, Guangdong 510640, China; 2 Department of Chemical & Biomolecular Engineering and Polymer Program, Institute of Materials Science, University of Connecticut, Storrs, Connecticut 06269, United States

## Abstract

Millions of tons of wastewater containing both inorganic and organic pollutants are generated every day, leading to significant social, environmental, and economic issues. Herein, we designed a graphene foam/TiO_2_ nanosheet hybrid, which is able to effectively remove both chromium (VI) cations and organic pollutants simultaneously. This graphene foam/TiO_2_ nanosheet hybrid was synthesized via a facile single-step one-pot hydrothermal method. The structure of the hybrid was characterized by scanning electron microscopy (SEM) and transmission electron microscopy (TEM). The hybrid was evaluated for both chromium (VI) and organic pollutants (using methyl blue (MB) as an example) removal, and the removal mechanism was also investigated. During water treatment, graphene and TiO_2_ nanosheets function complimentarily, leading to a significant synergy. The hybrid exhibited outstanding chromium (VI) and MB removal capacity, much superior to the performance of the individual pure TiO_2_ sheets or pure graphene foam. The hybrid could also be easily separated after water treatment, and exhibited excellent recycle stability. Considering the very facile synthesis of this graphene foam/TiO_2_ nanosheet hybrid, and its excellent water treatment performance and recycle stability, such a hybrid is promising for large scale production for practical applications where both chromium (VI) cations and organic dyes are the main pollutants.

Environmental crisis has attracted much attention these decades. Millions of tons of wastewater are generated every day and discharges to the rivers and oceans. Under most circumstances, it contains both inorganic and organic pollutants^1^. One major part of inorganic pollutants is heavy metal ions, which tend to accumulate in living bodies and known to be toxic^2^,^3^,^4^. Some heavy metals such as cadmium, nickel and chromium, are considered to be carcinogenic^5^,^6^. For chromium ions, chromium (VI) is highly toxic while the chromium (III) is one of the basic elements we need in our bodies^7^. The US Environmental Protection Agency (EPA) recommends that the level of chromium in water should be lower than 0.1 mg/L^8^. It is important to remove the chromium (VI) in the water to the limit required.

There are several ways for heavy metal removal, including chemical precipitation, ion exchange, membrane filtration, electrochemical treatment, and adsorption^2^,^9^,^10^,^11^,^12^,^13^. Among these methods, adsorption is one of the most economical and facile approaches to remove heavy metal ions in the water^14^,^15^,^16^. Some of the adsorbents can be regenerated and recycled due to the reversible nature of adsorption. Chromium (VI) removal by adsorption has been widely studied. Lo *et al*. used activated carbon as the adsorbent to remove chromium (VI) in water through van der waals interactions^17^. Alternatively, Hu and coworkers adopted maghemite nanoparticles as the adsorbent to remove chromium (VI) under acid condition based on the electrostatic interactions between the adsorbent and chromium (VI) ions^18^. Cao *et al*. synthesized nitrogen doped magnetic carbon nano-adsorbents that can effectively remove chromium (VI) via electrostatic attraction and redox reaction^19^. Furthermore, Guo *et al*. removed chromium (VI) from water through reduction using magnetic carbon nanocomposites derived from cellulose^20^. All adsorbents can adsorb chromium (VI) ions on their surface and eventually become saturated. In comparison, a much higher chromium (VI) removal efficiency can be achieved if the adsorbent can reduce the chromium (VI) to chromium (III). In this way, the adsorbent would never be saturated with chromium (VI) and can remove chromium (VI) continuously. Titanium dioxide, one of the most widely used photo-catalysts, has the ability to reduce chromium (VI) to chromium (III) under UV radiation^21^,^22^. Moreover, TiO_2_ is desirable because of its high efficiency, low cost, nontoxicity, and high stability^23^,^24^,^25^,^26^. Nanoscale TiO_2_ with a high specific surface area would be a particularly ideal choice for chromium (VI) removal.

Waste water also contains varies of organic pollutants, including pesticides, herbicides, phenols, and dyes. Those pollutants could be removed by oxidation, ion exchange, electro-dialysis, electrolysis, adsorption, etc. ref. 27. Adsorption is considered one of the best methods to remove organic pollutants, because of its universality, as well as low cost and ease of operation. Carbon based materials (such as activated carbon, carbon nanotubes, etc.) have been used broadly as adsorbents owing to their high specific surface area^28^,^29^. Graphene is a single atomic layer of graphite, which is composed of *sp*^*2*^-hybridized carbon atoms. Graphene has gained significant attention due to its incredibly high specific surface area (theoretical value: 2620 m^2^g^−1^), high thermal conductivity, and outstanding electrical conductivity^30^,^31^. Most of the pollutants are aromatic, which contain benzene rings and can be easily adsorbed on graphene surface due to π-π stacking effect^32^. Zhao *et al*. synthesized sulfonated graphene that adsorbed aromatic pollutants efficiently^33^. TiO_2_ can help degrade many kinds of organics under UV light^34^,^35^,^36^. Degradation reaction would happen after organic pollutants are adsorbed on graphene if TiO_2_ is attached on graphene.

Since wastewater contains both inorganic and organic pollutants, it is ideal if they can be removed through a single process. To achieve this goal, herein we designed a nanostructured hybrid, graphene foam attached with TiO_2_ nanosheets (graphene foam/TiO_2_ nanosheet hybrid, abbreviated as G/TiO_2_ hybrid) via a single-step one-pot hydrothermal method. For chromium (VI) removal, TiO_2_ nanosheets can reduce hexavalent chromium to trivalent chromium. During this process, graphene can promote the electron mobility and render the light excited electron-hole pairs on TiO_2_ nanosheets to separate more efficiently^37^. As a result, photocatalytic efficiency would be increased and therefore chromium (VI) removal rate would be enhanced. Moreover, G/TiO_2_ hybrid is able to effectively remove organic pollutants by using TiO_2_ to degrade the ones adsorbed on graphene foam with the assistance of UV radiation. In addition to serving as a platform to help collect organic pollutants, graphene foams can improve electron transfer rate and thus expedite the degradation process. As such, graphene foam and TiO_2_ nanosheets are complimentary to each other during both organic and inorganic pollutants removal. Furthermore, by designing a foam based structure, the hybrid can maintain a high removal efficiency owing to the high transport nature of the porous foam structure, as well as be easily separated from wastewater after treatment, leading to a facile and effective recycling process.

## Experimental

### Materials

Graphite (Grafguard 160–50N) was obtained from GrafTech. Sulfuric acid (95.0–98.0%), phosphorus pentoxide (>98%), potassium persulfate (>99.0%), potassium permanganate (>99.0%), titanium *n*-butoxide (>99%), hydrofluoric acid (48–51%), and potassium dichromate (>99.0%) were purchased from Alfa Aesar and used as received without further purification.

### Synthesis of graphene foam

A sample of 100 mg GO (made by the modified Hummers’ method^38^,^39^) was charged into a beaker with 20 mL DI water. After ultrasonication (Branson 8510R-MT, 250 W, 44 kHz) for 3 hours, a stable GO suspension was obtained. Then the GO suspension was transferred into a 50 mL Teflon^®^ lined autoclave and heated at 180 °C for 24 hours. After hydrothermal treatment, the suspension was filtrated and washed with DI water for 3 times, then dried under vacuum at room temperature for 24 hours^40^,^41^.

### Synthesis of TiO_2_ nanosheets

A sample of 1.6 mL titanium *n*-butoxide was added into a Teflon^®^ beaker together with 0.2 mL HF acid (48–51%). After stirring for 3 minutes, the solution was transferred into a Teflon^®^ lined autoclave and hydrothermally treated for 24 hours at 180 °C. After the hydrothermal treatment, the dispersion was filtrated and washed with DI water for 3 times, then dried under vacuum at room temperature for 24 hours^42^.

### Synthesis of G/TiO_2_ hybrid

A sample of 100 mg GO (made by the modified Hummers’ method^38^,^39^) was charged into a Teflon^®^ beaker with 20 mL DI water. After ultrasonication (Branson 8510R-MT, 250 W, 44 kHz) for 3 hours, 0.8 mL titanium *n*-butoxide and 0.1 mL HF acid (48–51%) were added into the GO dispersion during stirring. After stirring for 3 minutes, the dispersion was transferred into a Teflon^®^ lined autoclave. Then, the hydrothermal treatment was conducted for 24 hours at 180 °C. After the hydrothermal process, the suspension was filtrated and washed with DI water for 3 times, then dried under vacuum at room temperature for 24 hours.

### Characterization

The morphology and structure of the samples were characterized by scanning electron microscopy (SEM, JEOL JSM-6335F FESMs with an accelerating voltage of 10 kV) and transmission electron microscopy (TEM, FEI Tecnai T12 with an accelerating voltage of 120 kV).

### Evaluation of chromium (VI) removal ability

In this work, we choose fluorescent light as the light source for sample treatment out of the consideration of future practical applications. The ultraviolet radiation in fluorescent light is able to initiate the photocatalytic reaction for the removal of pollutants. A sample of 30 mg G/TiO_2_ hybrid was added into 25.0 mL (400 μg/L) potassium dichromate solution. After 4 hours of vigorous shaking under fluorescent light (SYLVANIA T5 fluorescent lamp, 28 W, 2 meters distance between the sample and lamp), 10.0 mL solution was collected and mixed with 0.2 mL phosphoric acid (85 wt%) and 0.5 mL coloring agent (1,5-diphenylcarbazide, DPC, 2.0 g/L)^43^. The obtained solution was analyzed by a UV-Vis spectrophotometer (Varian Cary 5000 UV-Vis NIR) by recording the absorbance. The absorbance of the characteristic peak at 540 nm is proportional to the concentration of chromium (VI) ion^44^ according to the Lambert-Beer’s Law. After the first cycle of evaluation, the G/TiO_2_ hybrid was collected by 2 minutes of gravity settling and subsequently washed by DI water for 3 times and dried in an oven. The dried G/TiO_2_ hybrid was used for the next cycle of evaluation through the same procedures.

### Evaluation of MB removal ability

A sample of 30 mg G/TiO_2_ hybrid was mixed with 25.0 mL (10 mg/L) methyl blue (MB) solution. After 4 hours of vigorous shaking under fluorescent light, 5.0 mL solution was collected and subsequently diluted by 10.0 mL DI water. The obtained solution was analyzed by a UV-Vis spectrometer (Varian Cary 5000 UV-Vis NIR) by recording the absorbance. The absorbance of characteristic peak at 664 nm is proportional to the concentration of MB^45^ according to the Lambert-Beer’s Law. After the first cycle of evaluation, the G/TiO_2_ hybrid was collected by gravity settling and subsequently washed by DI water for 3 times and dried in an oven. The dried G/TiO_2_ hybrid was used for the next cycle of evaluation through the same procedures.

## Results and Discussion

### Structure and morphology of G/TiO_2_ hybrid

**Figure 1:**
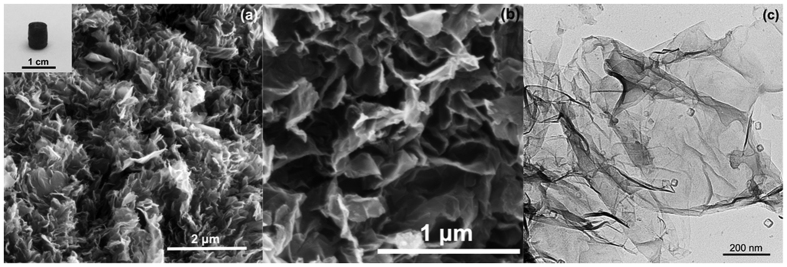
SEM (**a** and **b**) and TEM (**c**) images of G/TiO_2_ hybrid. The inset in (**a**) shows a digital image of the prepared G/TiO_2_ hybrid.

Graphene can be synthesized as a form of foam via hydrothermal treatment of graphene oxide suspension^40^,^41^. G/TiO_2_ hybrid was synthesized via a similar approach by incorporating TiO_2_ nanosheets. The morphology and structure of the G/TiO_2_ hybrid was characterized by SEM and TEM. As presented in Fig. 1a and b, the G/TiO_2_ hybrid shows a rough surface and a porous structure. After the hydrothermal reaction, the graphene oxide and TiO_2_ nanosheets were condensed to a small cylinder-shaped foam, as shown in the inset of Fig. 1a. Under TEM, square-shaped TiO_2_ nanosheets were uniformly dispersed on the surface of large graphene sheets, as shown in Fig. 1b. Graphene sheets also exhibited folds and wrinkles. The exact amount of graphene and TiO_2_ in the synthesized hybrid was determined by thermogravimetric analysis to be 23.6 and 6.4 mg, respectively.

### Hexavalent chromium removal ability of G/TiO_2_ hybrid

**Figure 2:**
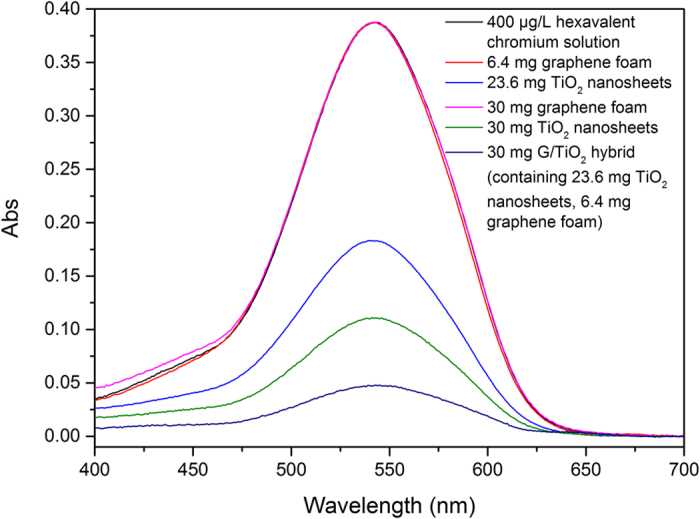
UV-Vis spectra of 400 μg/L chromium (VI) solution after being treated by different samples.

Figure 2 shows the chromium (VI) removal evaluation results by the G/TiO_2_ hybrid. Pure graphene foam and pure TiO_2_ nanosheets were also synthesized and evaluated as controls. The results show that G/TiO_2_ hybrid can remove chromium (VI) ions much more effectively compared to the two control samples. Pure graphene foam has virtually no adsorption ability, which is expected. This also indicates that TiO_2_ is the key component in the G/TiO_2_ hybrid for chromium (VI) removal. Meanwhile, the results clearly show that the performance of the pure TiO_2_ nanosheets is much inferior to that of the graphene/TiO_2_ foam that contains the same amount of TiO_2_ nanosheets, which is exactly as designed. This series of results show that the G/TiO_2_ hybrid exhibited the designed synergy between the graphene foam and TiO_2_ nanosheets. Graphene is a single layer of graphite composed of *sp*^*2*^-hybridized carbon atoms, which possesses superior electron conductivity^31^. Since the entire removal process is basically a photocatalytic reduction of chromium (VI) ions, the existence of graphene can expedite the separation rate of electron-hole pairs by rapidly conducting the light-excited electrons^37^. In this way, the photocatalytic efficiency is improved significantly, thus G/TiO_2_ hybrid demonstrates a much higher chromium (VI) removal rate than the pure TiO_2_ sheets.

To further validate the efficiency of the G/TiO_2_ hybrid for chromium (VI) removal, it was also evaluated with the same mass amount of TiO_2_ nanosheets. As shown in Fig. 2, the chromium (VI) removal ability of 30 mg of G/TiO_2_ hybrid (containing 23.6 g TiO_2_ nanosheets) was even higher than that of 30 mg of pure TiO_2_ nanosheets. This result further suggests that the existence of graphene can effectively speed up the reduction of chromium (VI) ions, thus increasing the practical capacity of TiO_2_ nanosheets for chromium (VI) removal.

**Figure 3:**
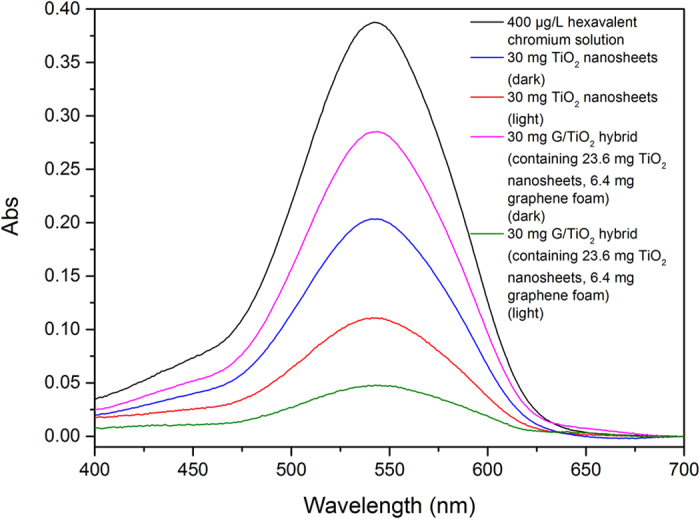
UV-Vis spectra of 400 μg/L chromium (VI) solution after treatment with TiO_2_ sheets and G/TiO_2_ hybrid in fluorescent light and in dark.

As discussed above, TiO_2_ nanosheets can barely adsorb chromium (VI) ions physically; the main mechanism for chromium (VI) removal by TiO_2_ nanosheets is photo-catalyzed reduction of hexavalent chromium to trivalent chromium under UV radiation^21^,^22^. As such, we further evaluated the chromium (VI) removal efficiency of the TiO_2_ sheets under both fluorescent light and dark. The TiO_2_ nanosheets only removed a small amount of chromium (VI) ions under the dark condition, while it exhibited a much higher adsorption under the fluorescent light, as shown in Fig. 3. This result is expected and is consistent with our initial hypothesis. Figure 3 also shows the chromium (VI) removal capacity of the G/TiO_2_ hybrid under fluorescent light and dark. The 30 mg G/TiO_2_ hybrid removed less chromium (VI) ions under the dark compared to the 30 mg of pure TiO_2_ nanosheets under the same conditions, which is probably because of the fact that there is only ca. 23.6 mg of TiO_2_ nanosheets in the hybrid, and such TiO_2_ nanosheets are less exposed to light, and meanwhile graphene cannot adsorb chromium (VI) ions. After applying room light, the removal capacity of the G/TiO_2_ hybrid (containing ca. 23.6 mg of TiO_2_ nanosheets) increased dramatically, even higher than that of the 30 mg of pure TiO_2_ nanosheets. This result further shows the promoting effect of graphene on TiO_2_ nanosheets for chromium (VI) ions removal, in which graphene can increase the electron mobility^37^. As such, graphene can improve the photocatalytic efficiency of TiO_2_ nanosheets and therefore chromium (VI) removal rate can be significantly enhanced.

**Figure 4:**
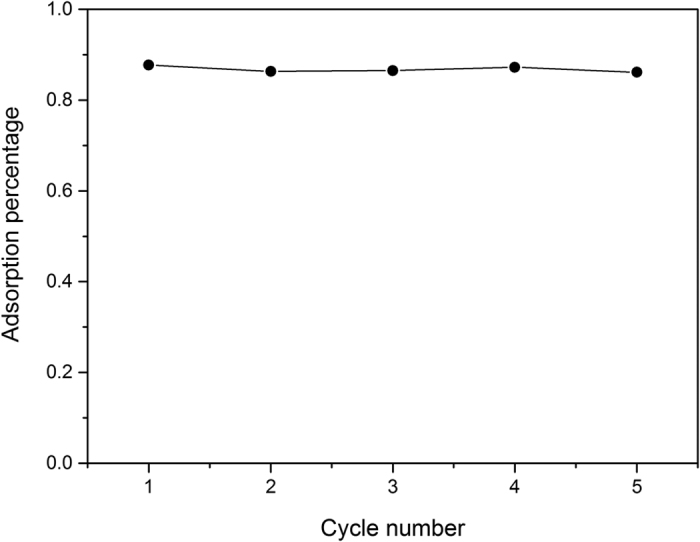
Recycle test of chromium (VI) removal using the G/TiO_2_ hybrid.

The recycle evaluation result in Fig. 4 shows that the G/TiO_2_ hybrid has an excellent recycle stability for chromium (VI) removal. The removal capacity in terms of percentage of chromium (VI) ions removed changed little after 5 cycles of operation. Since the adsorption mainly due to the photocatalytic degradation of chromium (VI) and limited physical adsorption of chromium (VI) ions on the G/TiO_2_ hybrid, it is able to maintain excellent recycle stability.

### Methyl blue removal ability of G/TiO_2_ hybrid

**Figure 5:**
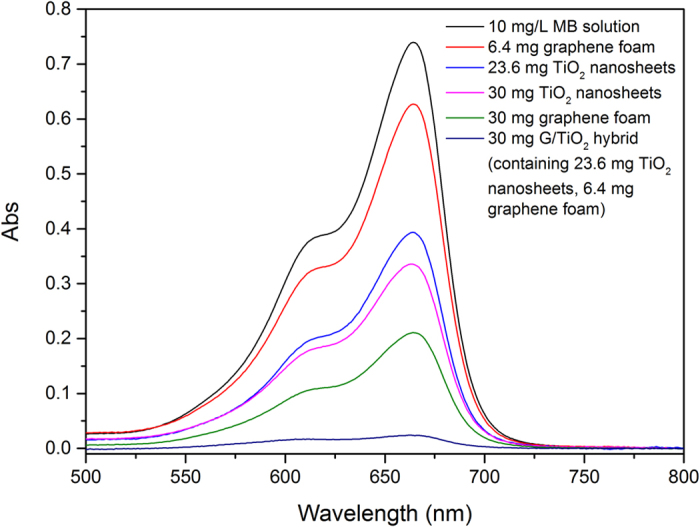
UV-Vis spectra of 10 mg/L MB solution after treated by different samples.

Methyl blue (MB) was selected as a representative organic component to evaluate the organic pollutant removal capacity of the G/TiO_2_ hybrid. The MB removal capability of 30 mg of G/TiO_2_ hybrid (containing ca. 6.4 mg of graphene foam and 23.6 mg of TiO_2_ nanosheets) was proved to be much higher compared to 6.4 mg of pure graphene foam only or 23.6 mg of pure TiO_2_ nanosheets only, as shown in Fig. 5. Moreover, 30 mg of G/TiO_2_ hybrid removed more MB than 30 mg of pure graphene foam only or 30 mg of pure TiO_2_ sheets only, clearly showing the synergy between the two components.

Graphene foam is composed of graphene sheets. Since graphene sheets can adsorb organics, especially the ones with benzene rings due to π-π stacking effect, MB could be physically adsorbed by graphene foams. TiO_2_ nanosheets can remove MB by photocatalytic degradation. This photocatalytic reaction requires photons to excite the electrons and further react with MB. The rate of the photocatalytic reaction could not meet the rate of physical adsorption of graphene. This explains why the pure graphene foam can remove more MB from solution compared to the pure TiO_2_ nanosheets (as shown in Fig. 5). The G/TiO_2_ hybrid contains TiO_2_ nanosheets attached on the surface of graphene. Under such a structure, graphene foam can adsorb MB to the surface and facilitate TiO_2_ nanosheets to be in contact with MB. As a result, photocatalytic reaction occurred and the adsorbed MB was degraded, which opens space for additional MB molecules to be adsorbed by graphene foam and subsequently degraded by TiO_2_ sheets. In addition, graphene can also help speed up the electron transfer rate in the photo-catalysis process^37^. Such coherent synergy between the physical adsorption by graphene sheets and the photo-catalyzed reduction by TiO_2_ nanosheets lead to very effective MB removal by the G/TiO_2_ hybrid.

**Figure 6:**
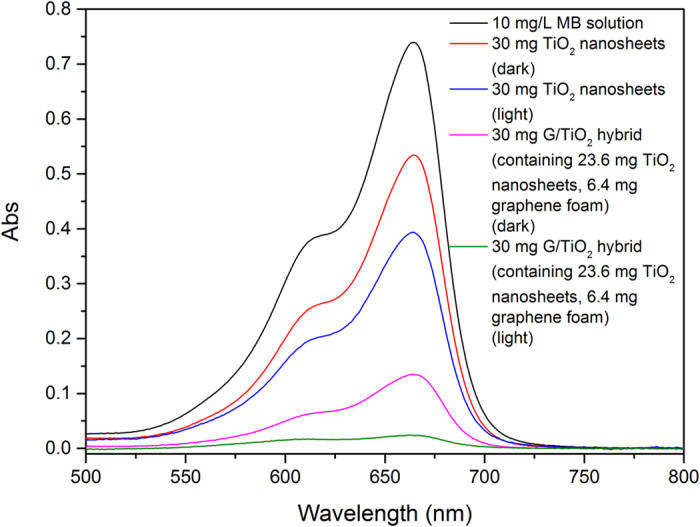
UV-Vis spectra of 10 mg/L MB solution after treated with pure TiO_2_ sheets and G/TiO_2_ hybrid in fluorescent light and in dark.

To further investigate the photo-catalyzed degradation, the MB removal reaction was conducted under both fluorescent light and dark. The results showed that TiO_2_ nanosheets can remove a small amount of MB even at dark (Fig. 6), which suggests that TiO_2_ nanosheets can remove MB by physical adsorption as well. However, the overall MB removal capacity is relatively low. The result of MB removal capacity of the G/TiO_2_ hybrid under room light and dark shows that a large percentage of MB molecules were removed by physical adsorption of both TiO_2_ nanosheets and graphene foam while not applying the light. This is mainly contributed by the high physical adsorption capacity of graphene foam. However, nearly all MB molecules were removed when applying light. This result again confirms that the MB removal by G/TiO_2_ hybrid is a combination of physical adsorption and photocatalytic degradation. TiO_2_ can degrade MB while graphene can physically adsorb MB to its surface until saturated. For graphene/TiO_2_ foam, the MB adsorbed on graphene foam can be degraded by TiO_2_ nanosheets. Thus, graphene surface would not be saturated and thus can adsorb MB continuously. Meanwhile, TiO_2_ nanosheets can degrade MB more rapidly with the help of graphene, since graphene can help improve electron transfer rate. As a result, there is an ideal synergistic effect between TiO_2_ and graphene, leading to highly effective removal of MB.

Recycling is essential for adsorbents. It would be ideal if adsorbents can be used for many times while the efficiency remains high. The G/TiO_2_ hybrid was designed to be used for multiple cycles because of its removal mechanism. Its overall foam structure also makes it easy to be separated from the water being treated.

**Figure 7:**
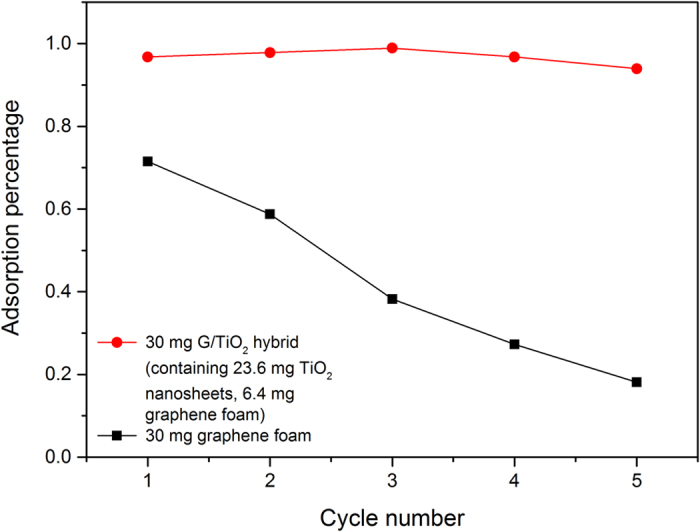
Recycle test of MB removal using G/TiO_2_ hybrid and graphene foam only.

The recycle testing result (Fig. 7) shows that the G/TiO_2_ hybrid has a very good recycle stability, while graphene foam lost part of adsorption ability after each cycle. This result is expected and can be explained by the different adsorption mechanisms of these two foams. Pure graphene foam can only physically adsorb MB to its surface, thus eventually will be saturated during the treatment. As a result, it will lose some MB removal ability after each cycle. For the G/TiO_2_ hybrid, it is never the case. TiO_2_ nanosheets can degrade the MB molecules adsorbed by graphene foam. In this way, graphene surface would always have open room for MB molecules, thus the adsorption process goes on and on.

### Hexavalent chromium ions & methyl blue mixture removal ability of G/TiO_2_ hybrid

**Figure 8:**
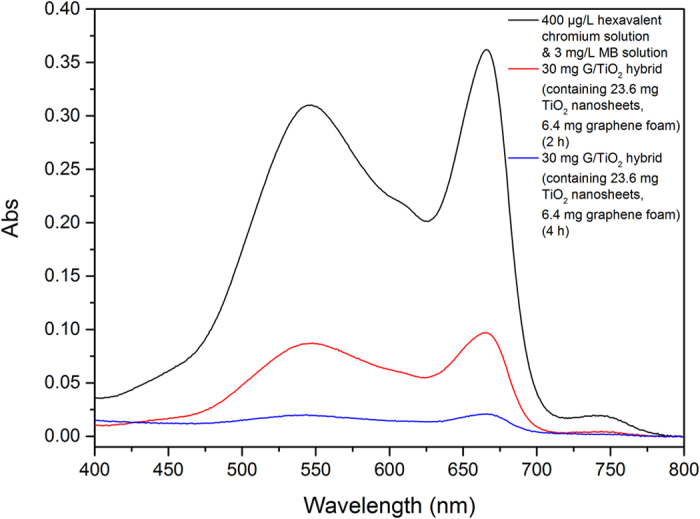
UV-Vis spectra of a mixture of hexavalent chromium and MB after 2 and 4 hours of treatments by the G/TiO_2_ hybrid.

As most waster water contains both inorganic and organic pollutants, the synthsized G/TiO_2_ hybrid was also evaluated for simultaneous removal of chromium (VI) ions and MB. Figure 8 displays the mixed adsorption of both pollutants by the G/TiO_2_ hybrid. The results show that the G/TiO_2_ hybrid can effectively remove both chromium (VI) ions and MB simultaneously and effectively. This results suggests that the G/TiO_2_ hybrid are promising for practical applications where both chromium (VI) ions and organic pollutants present.

## Conclusions

In this work, a G/TiO_2_ hybrid was designed and synthesized through a single-step one-pot hydrothermal method. The hybrid exhibited excellent chromium (VI) and methyl blue (MB) removal ability compared to pure TiO_2_ sheets or pure graphene foam. During water treatment, graphene foam and TiO_2_ nanosheets function complimentarily, leading to a significant synergy. The hybrid could also be easily separated after water treatment, and exhibited excellent recycle stability. Considering the very facile synthesis of this G/TiO_2_ hybrid, and its excellent water treatment performance and recycle stability, such a hybrid is promising for large scale production for practical applications where both chromium (VI) cations and organic dyes are the main pollutants.

## Additional Information

**How to cite this article:** Wang, W. *et al*. Single-step One-pot Synthesis of Graphene Foam/TiO_2_ Nanosheet Hybrids for Effective Water Treatment. *Sci. Rep.* **7**, 43755; doi: 10.1038/srep43755 (2017).

**Publisher's note:** Springer Nature remains neutral with regard to jurisdictional claims in published maps and institutional affiliations.
